# Supplementary material for: Empirical methods for controlling false positives and estimating confidence in ChIP-Seq peaks
Source: BMC Bioinformatics. 2008 Dec 5;9:523. doi: 10.1186/1471-2105-9-523 (PMC2628906; doi:10.1186/1471-2105-9-523)
Supplement: Additional File 1 — A variety of html documents from the USeq web site detailing the available applications, their best usage, output file type descriptions, command line menus, etc. [file 1471-2105-9-523-S1.zip › USeqUserGuides/JavaDoc.zip!/JavaDoc/util/bio/parsers/gff/class-use/Gff3Feature.html]

Uses of Class util.bio.parsers.gff.Gff3Feature


|  |  |  |
| --- | --- | --- |
| **Overview** \| **Package** \| **Class** \| **Use** \| **Tree** \| **Deprecated** \| **Index** \| **Help** | |  |
| PREV   NEXT | **FRAMES**    **NO FRAMES**     **All Classes** |  |


---


## **Uses of Class util.bio.parsers.gff.Gff3Feature**

| Packages that use Gff3Feature | |
| --- | --- |
| **util.bio.annotation** | For representing gene annotation. |
| **util.bio.parsers.gff** | GFF2 and GFF3 parsers. |

Uses of Gff3Feature in util.bio.annotation

| Methods in util.bio.annotation with parameters of type Gff3Feature | |
| --- | --- |
| `boolean[]` | `ExportIntergenicRegions.maskBoolean(Gff3Feature[] features)`             Uses a boolean[] to score whether a base is part of an annotation (true) or not (false). |

Constructors in util.bio.annotation with parameters of type Gff3Feature

`ExonIntron(Gff3Feature f)`

`GenericFeature(Gff3Feature f)`

`Transcript(Gff3Feature f)`

`Translation(Gff3Feature f)`

Uses of Gff3Feature in util.bio.parsers.gff

| Methods in util.bio.parsers.gff that return Gff3Feature | |
| --- | --- |
| `Gff3Feature[][]` | `Gff3Parser.getChromSplitFeatures()`             Returns the features split by chromosome. |
| `Gff3Feature[]` | `Gff3Parser.getFeatures()` |

| Methods in util.bio.parsers.gff with parameters of type Gff3Feature | |
| --- | --- |
| `void` | `DmelRel4Extractor.buildGeneGrp(Gff3Feature f)`             Creates a new gene group, call this last after having loaded the exons, transcripts, and translations. |
| `void` | `DmelRel4Extractor.buildGenericFeature(Gff3Feature f)` |
| `static int[][]` | `ParseWormBaseGffToUCSC.mergeFeatures(Gff3Feature[] features)`             Uses a boolean[] to score whether a base is part of an annotation (true) or not (false). |
| `static void` | `Gff3Feature.saveGFF3TextFile(Gff3Feature[] g, java.io.File newGFF3File)`             Prints an array of GFF3Features as a plain text file |

---


|  |  |  |
| --- | --- | --- |
| **Overview** \| **Package** \| **Class** \| **Use** \| **Tree** \| **Deprecated** \| **Index** \| **Help** | |  |
| PREV   NEXT | **FRAMES**    **NO FRAMES**     **All Classes** |  |


---
